# Preclinical research (on rare diseases): we need to talk about health equity

**DOI:** 10.1007/s00335-024-10080-1

**Published:** 2024-10-26

**Authors:** Andy Greenfield

**Affiliations:** Nuffield Department of Women’s & Reproductive Health, Institute of Reproductive Sciences, University of Oxford, Alec Issigonis Way, Building 8000, OX4 2HW Oxford, UK

**Keywords:** Preclinical models of disease, Animal models, Organoid models, Precision medicine and care, Ethics of healthcare equity, Ethical review

## Abstract

There is a thriving, worldwide, biomedical research community working to understand the molecular bases of diseases of all types, continuously driving improved diagnostics and therapies. Developments in genetics and experimental medicine are yielding novel genetic therapies that were hardly dreamt of 40 years ago. But along with these scientific achievements, there exist challenges in ensuring that 21st century medical interventions are accessible to all who need them. This perspective will discuss how preclinical research, with a focus on rare diseases, can better contribute to healthcare ecosystems that are oriented towards greater health equity. This contribution may require changes to the prevailing scientific research culture that will need support from relevant institutions and the wider community.

## Introduction

In March 2023, I attended the 3rd International Summit on Human Genome Editing in London. This was, thankfully, a less dramatic and incendiary affair than the 2nd Summit in Hong Kong in 2019, at which a scientist from China reported the birth of twins following genome editing and uterine transfer of human embryos. The London Summit was notable for pivoting away (to some extent) from discussions of heritable uses of genome editing for disease prevention and towards therapeutic uses in affected individuals. One highlight was a talk given by Victoria Gray, the first person to receive a genome editing-based treatment for sickle cell disease as part of a clinical trial (Singh et al. 2024). She spoke movingly (and bravely) about her experiences of living with sickle cell disease and the remarkable improvement in her condition following treatment.

This anecdote is chosen with a purpose. It neatly encapsulates so many issues, scientific and social, surrounding contemporary biomedical research and clinical application. Ms Gray is a woman of colour. This is no coincidence: sickle cell disease is most prevalent in sub-Saharan Africans and black Americans. More than 75% of people with the disease are born in sub-Saharan Africa and India. But this observation raises the question of whether the very people that most need new interventions to improve their health can get access to them. What roles are played (and have been played historically) by ethnicity and geographical location in such accessibility and associated health disparities? (Answer: significant ones (National Academies of Sciences, 2017). Has biological sex – and sex bias in research – also played a role and does it continue to do so? (Answer: yes (Avery and Clark 2016; Patwardhan et al. 2024)). Clinical trials, such as the one she consented to, are highly expensive to design and perform and, perhaps as a consequence, their status as the ‘gold standard’ of medical evidence is frequently contested. Victoria Gray is a reminder that the development of new technologies and the translation of preclinical research into innovative therapies are events that occur (or do not) in particular social and political circumstances, as well as scientific eras. Their success or otherwise is a multi-factorial matter.

For someone who studied and began to practice molecular genetics in the 1980s, it seems a near miracle that such a treatment was not only conceived but was also successful (to our knowledge – it is early days for the use of such interventions). But we must now dream, not just of *technological* breakthroughs that can deliver such an obvious good to someone (although we still desperately need these), but also of breakthroughs in developing a research and clinical translation ecosystem that can deliver high-quality, innovative healthcare *equitably*, i.e. to all those who need it, on a global scale. Ms. Gray’s treatment was the product of decades of research, fundamental and clinical, public and private, characterised by certain institutional and funding structures; of a healthcare system that provides new treatments to those who need them only after rigorous assessment processes (that can sometimes be flawed) and only if they are deemed affordable; and what is affordable can vary wildly from individual to individual, community to community and from state to state. Is it an ecosystem that is fit for purpose? New gene therapy and genome-editing based therapies are renowned for their astronomical costs and so raise the topic of distributive justice because healthcare budgets are finite and under pressure. A session at the London Summit on affordability and equity in provision of genome editing-based interventions suggested that this dimension, particularly the global provision of high-tech genetic interventions, is a challenge that some think we cannot meet. It is reassuring, therefore, that others do not agree (Harlow and Adair 2024; Kliegman et al. 2024).

This perspective considers, in particular, the role that could (should) be played by preclinical research in promoting (by orienting itself towards) a healthcare ecosystem characterised by equity (and justice), with a focus on rare diseases. By the very nature of perspectives, it does not aim to be systematic and comprehensive. Instead, I will discuss some issues that strike me as important based on personal experiences and a reading of the recent literature on this topic and suggest some approaches that could be adopted in preclinical research, without dwelling on the details of regulation and governance frameworks, which, whilst vital, vary so much from nation to nation.

## The ambition of health equity

We can begin by making a distinction between equity and equality. Equality amounts to everybody having, or at least being entitled to, the same – the same amount of anything, the same quality of anything, etc. Equity exists when everybody receives (or is entitled to) whatever it is they *need*. What is needed, of course, varies from individual to individual, from community to community or from state to state. Health equity has been defined as follows: “Health equity means that everyone has a fair and just opportunity to be as healthy as possible. This requires removing obstacles to health such as poverty, discrimination, and their consequences, including powerlessness and lack of access to good jobs with fair pay, quality education and housing, safe environments, and health care” (Braveman 2017). This definition, whilst no doubt contestable, makes it clear that there are a number of determinants of health, including not just biological but also social and political factors. The evidence suggests that many health outcomes are affected by social, economic and environmental factors: poverty (of different types), socioeconomic class, and ethnicity are all associated with poorer health outcomes. Problems like unemployment, poor transportation, poor housing, exposure to violence and the deterioration of neighbourhood environments (both social and physical) all contribute to health outcomes and they do so unequally. Roughly, unequal distribution of the determinants of health results in inequitable distribution of the benefits of good health and the burdens of ill-health. To give just one example, from the US: African American women have a higher chance of giving birth to low-birthweight offspring and their newborns have higher infant mortality rates (National Academies of Sciences, 2017). Numerous other instances of such health disparities are well documented (Macias-Konstantopoulos et al. 2023).

### Rare diseases and health equity

Addressing the unequal distribution of health determinants is a matter of social change, and science is a social activity that can play a role. The topic here is how preclinical research can contribute to the aim of health equity, with an emphasis on rare diseases (RDs). Whilst there is no universal definition of RDs, they are commonly defined as those affecting fewer than or equal to 1 in 2000 individuals in the population. There are estimated to be around 6–8,000 RDs, affecting 3.5 million people in the UK and over 300 million globally (Nguengang Wakap et al. 2020). They are, therefore, individually rare but collectively common. Rare *genetic* diseases have benefitted from the rise of genomics and the improved ability to identify causal variants through exome/genome sequencing programmes and supporting studies using model systems. But many remain undiagnosed. It is clear that, as with other health determinants, the benefits of modern genetic diagnostics are not available to all.

It is also important to note that RDs present a unique challenge to the ambition of health equity, since some relevant difficulties arise *precisely* due to the rareness of these conditions, including the potential difficulty in diagnosis, inadequate clinical management and very limited access to treatments, development of which is hindered by ignorance of the underlying mechanisms of disease and the difficulty of implementing clinical trials (Halley et al. 2022, 2023; Adachi et al. 2023). Further research is required to better diagnose and understand rare diseases, but such research requires funding. Even here, RDs present a challenge, since companies developing innovative therapies may not invest in RDs due to the small market size. The role of philanthropy in funding research, driven by advocacy on behalf of RD sufferers and their families, also highlights the structural complexity of health disparities in this context, since not all communities and geographical regions are equally likely to have the social capital to produce eloquent and influential advocates for RD research or philanthropists who can invest in such research.

Finally, diversifying recruitment and enrolment into the genome sequencing programmes that fuel preclinical research is required to ensure that the needs of underserved or vulnerable populations are met (Baynam et al. 2017; Serrano et al. 2023; Gaviglio et al. 2023). All these observations suggest an ethical imperative for the scientific community to do even more to reduce the burden on all RD sufferers and their families by speeding up diagnosis and improving care through the provision of novel treatments. This is especially challenging in low-middle income countries (LMICs), where collaborative strategies are required (Tkemaladze 2024).

What follows are some suggestions, mostly not novel, for how preclinical researchers, including those developing models to examine the genetic bases of rare diseases, might respond to the imperative of making preclinical research better aligned with the promotion of health equity, ultimately leading to better diagnostics, novel treatments and more effective healthcare solutions for all.

## Ensure study populations are diverse

The use of animal models is key to better understanding the role played by genetic variants in human disease, sometimes being vital in confirming the causal role of a gene/variant in a disease phenotype. Such models, notably using the mouse, also act as important tools for assessing the feasibility of novel therapies (Koblan et al. 2021; Reichart et al. 2023). But animal research is not without its critics. I have discussed ethical issues in the context of using genome editing in research animals elsewhere and will not repeat those arguments here (Greenfield 2017). Briefly, it is vital that animals are only used when alternatives cannot deliver the necessary scientific insights and that suitable models (species, genetic background, choice of variant(s) etc.) are used, depending on research aims, in accordance with the principles of the 3Rs. It is even more challenging to make a judgment about suitability now, given the rapid progress of human organoid (Liu et al. 2024) and assembloid (Meng et al. 2023) approaches and related stem cell-based developments, such as human embryo models (Zernicka-Goetz 2023).

Whatever models are used, and insights into the systemic, physiological origins of certain diseases will continue to require animals for the forseeable future, it is important that the genetic variants being modelled represent the full diversity of human genomes and disease-associated variants. It is well established that human sequencing programmes must aim to capture the full range of human genetic diversity if the knowledge they yield, and the diagnostic and therapeutic opportunities such knowledge delivers, are to be relevant to the full range of human subjects that seek or have an interest in such interventions. The same point applies to the preclinical research models (human cell lines, organoids etc.) that are used to investigate the relationship between gene variants and phenotypes and to assess novel therapeutics: they should be representative of diverse human communities too, with distinct genetic architectures underlying disease. This is the case even if therapies target functional elements commonly affected by distinct upstream mutations in a key biochemical pathway, since confirmation that such an approach can rescue a disease phenotype in diverse genetic models is required.

It has been argued that under-representation (or simplified grouping) of patients from minority groups in sequencing cohorts and associated preclinical models is also a major hurdle in addressing cancer disparities (Halmai and Carvajal-Carmona 2022). For example, epidemiological studies suggest that black men and women are twice as likely to die from prostate and breast cancer, respectively, than are white people. But the cancer genome atlas (TCGA) has been dominated by samples from individuals of white ancestry (Carrot-Zhang et al. 2020). Again, patient-derived organoids and patient-derived xenograft models (PDXs) should also reflect the diversity of cancer genomes in order to be effective in understanding and treating disease in diverse communities and human ancestries. In a similar vein, the use of genetic ‘humanised’ mice, containing a segment of DNA of human origin that can replace the endogenous murine equivalent, is of growing importance in assessing the causality and underpinning mechanisms of pathology of human gene variants, especially in neurobiology (Nair et al. 2019). Details of the human genomes that are the sources of human segments in such mice, including ancestry, should be routinely reported. Is the particular source justified? Are the sources representative of human genome diversity? Mouse geneticists understand that simply referring to a ‘mouse’ gene without specifying the strain of origin (or justifying its selection) would be unacceptable in a study and the same considerations apply to human genome data employed in such studies.

Aiming for inclusivity in preclinical research will inevitably be constrained by the economic realities of funding. It is unrealistic for individual research teams or even institutes to be as inclusive as is required to systematically address health disparities. One solution is for such an aim to be realised through global collaboration, such as international consortia, whereby research is coordinated across institutes and geographical regions to ensure inclusivity as an overall outcome. This should also involve greater support for the development of preclinical research in LMICs through international funding initiatives. Such collaboration should also be multi-disciplinary: the insights of life scientists, social scientists and bioethicists can and should be shared and useful synergies exploited.

## Engage under-represented communities

There is a slogan familiar to anybody that has worked on the governance of emerging biotechnologies in the medical space: ‘nothing about us, without us’. The point is that many individuals that are purportedly the focus of particular research programmes leading to potential clinical interventions do not wish to be mere subjects but also want to be ‘at the table’, contributing to the conception of research at an early stage in an attempt to ensure that preclinical research will ultimately address relevant health issues. Participation at early stages means not just through clinical interfaces (recruitment to sequencing programmes, consenting, etc.) but also when preclinical research, involving models such as animals and organoids, is conceived and planned. How many research institutes, where fundamental and applied preclinical research occurs, are *actively* reaching out to minority communities? Have they established appropriate fora for dialogue (and not just one-way education initiatives, although these remain important) about their research and the health needs of such communities, rather than relying on their clinical collaborators to do so? Establishing channels of communication and feedback at different points of the research pipeline will be key to informing existing and future research priorities. Such communication is also important precisely because much preclinical research presages clinical interventions that are highly innovative and often controversial, especially for communities that have historically been marginalised or exploited by mainstream medical research. Dialogue at early stages is a precondition of relationships worthy of *trust* developing between innovators and all patients/advocates/carers and broader publics with an interest in such innovation being successful.

It follows from the need to engage more widely that all relevant data should be made publicly available for scrutiny. Such data should include details of demographics used in preclinical studies, of the sex, age and genetic ancestry of human participants (and the DNA, cells and tissues they consent to donate) and how/why the data generated have led to additional, detailed functional studies in preclinical models in particular cases.

## Consider all diseases fairly

Prevalence is just one dimension of a disease. We should be careful not to erect impregnable taxonomic barriers between rare diseases and more common ones – since it is more accurate to see these as existing on a spectrum (Sergouniotis et al. 2023). Another dimension is the pathology associated with a disease, which can vary greatly in severity between individuals. The *seriousness* of a disease is commonly a consideration in decision-making in a wide range of areas (Kleiderman et al. 2024): the use of risky interventions and whether they are justified, funding allocations and even the likelihood of charitable donations are all likely to have seriousness – especially ‘life-threatening or not?’ – as a central consideration. But not all diseases are life-limiting. Approximately 1 in 6 people globally have experienced infertility at some point in their lives (WHO 2023). Infertility frequently causes stigmatisation of affected individuals and can cause great psychological harm. It is highly complex genetically and physiologically and, despite its prevalence, it is reasonable to view infertility as many rarer diseases with a common functional outcome. Is infertility a neglected disease? It often seems to be treated as exceptional or raises difficulties for health policy. For example, in its categorisation of heritable conditions that might in future justify the use of safe heritable genome editing (for their prevention), based on criteria including seriousness, the National Academies Commission on Heritable Human Genome Editing placed infertility in a ‘special category’ of its own, distinct from all other monogenic conditions (National Academy of Medicine, 2020). Assisted reproductive techniques, such as in vitro fertilisation and associated ‘add-ons’, often offered by profit-driven private companies, are increasingly associated with exploitation of vulnerable and often desperate patients (Lancet 2024). This desperation in itself should be a clear indication of the degree of suffering experienced by those living with infertility (and subfertility). It is in the spirit of health equity that we should recognise all pathologies and the impacts they have on people’s lives and not rely on an overly narrow conception of ‘serious’ to legitimise the development of interventions.

## Ethical review and cultures of ethical curiosity

Frameworks of ethical review are common in most countries, varying from law-based regulation to self-governance models. They cover the use of human cells, tissues and organoids as well as animals in research. This means that infrastructures of ethical oversight already exist in most countries, which is itself important. But a cultural shift may be just as important as the formal processes that occur under the auspices of institutional review boards and related bodies. I chaired an AWERB (an animal welfare ethical review body) in the UK for around a decade and I do not recall health equity being directly addressed at any point – although our discussions were wide-ranging, nevertheless, and often went beyond conventional harm-benefit analyses. It may be that an AWERB is not the *best* place for discussions about health equity, where the focus is rightly the welfare of research animals; but it would be reasonable to ask a researcher how or whether considerations of equity had been taken into account when choosing the particular models that were being scrutinised, since this would be relevant to a discussion about the wider impact of the research. Of course, such a question might reasonably also be asked in lab meetings, in seminars or works in progress discussions – anywhere, in fact. We should encourage ethical curiosity amongst scientists – ‘why should we do *this*, but not *this*?’ But it might require a shift in cultural expectations – for example, that a life sciences graduate student could be reasonably *expected* to be conversant in a topic like health equity and its relationship to preclinical research, along with other social justice themes, such as justifying their use of animals through the construction of arguments. A shift like this might take time and will require training. How common is training for life scientists in subjects such as bioethics, sociology and philosophy? Perhaps not common enough – although I have not seen an analysis of the relevant data. Might there be a social scientist ‘in residence’ at a biomedical research institute, occasionally, to act as a ‘critical friend’, discuss governance and society-oriented policy initiatives and generally aid with training in bioethics?

Health equity is compromised by geographic disparities, because access to relevant specialists and advanced diagnostics tends to be concentrated in urban areas or rich countries, leaving rural populations in LMICs underserved. I would contend that many early career scientists researching RDs in Europe and the US and other rich parts of the world would benefit from a period of research in an LMIC, learning about their research priorities, of course, but also the economic or cultural constraints that operate there - perhaps as part of an exchange programme that also allows LMIC researchers to experience research in richer countries, all funded through large international collaborations that prioritise LMIC interactions with richer nations (Shaaban et al. 2024). In this spirit, the WHO has promoted global programs of international collaboration in support of human genomics to address inequitable access to genomics (Ambrosino et al. 2024).

In addition to obvious changes to funding regimes, such a cultural shift might require a change in the expectation of what a life sciences PhD is *for*, for example. *Just* to generate publishable biological data? Or to also contribute to a wider debate about the role of science in society, about science as an ethical enterprise on the global stage? There are choices to be made. It may be that better orienting preclinical research towards greater health equity may require such cultural evolution; its ramifications on the research ecosystem – funding, the nature of research assessment exercises etc. - would be great. Imagine all journals and funders in this space requiring a ‘health equity statement’ to accompany submissions, and so on. The question of research ethics reviews in biomedical science and their legitimate *scope* – e.g. whether research ethics committees consider the long(er)-term societal implications of transformative research or the adoption of disruptive technologies and are appropriately constituted to do so - is also a topical one (Rothstein et al. 2024).

Finally, it is clear that scientific research should itself be something performed by suitably qualified women and men (and persons who identify as neither) of all ages, sexualities, ancestries and social backgrounds, if it is to (rightly) reflect the diverse societies in which it is embedded and which fund it. But diversity alone may not be enough to change cultures if young scientists do not feel that they can speak truth to power. Research institutes and universities should ensure that they create and support environments in which challenges, scientific and ethical, are the norm and nobody’s behaviour is beyond scrutiny.

## Conclusion

Health is an important part of human flourishing – of living a good life – and health equity is therefore an element in the broader topic of social justice, since a just society will promote flourishing for *all*. I assume here that I don’t need to mount an argument in favour of justice and will assume health equity is an incontestable social good. It is morally unconscionable that a large proportion of humanity suffers from diseases of poverty and hunger, of poor sanitation and food insecurity, whilst the other suffers from diseases often (but not exclusively) associated with excess – of obesity and associated comorbidities. Scientific research occurs with this miserable fact as its backdrop – but it is one that should also act as an incentive for scientists to strive even further.

The pursuit of a preclinical scientific research base that promotes health equity is something that relies on both an ethical ambition – the fair and just distribution of health – and a scientific one – the rapid translation of knowledge produced by fundamental and biomedical scientific research into improved diagnostics and effective therapeutics for all those that need them. These two go hand in hand, and so it is evident that one should not be used in opposition to the other. It is, however, possible for anti-science rhetoric (depressingly, all too common) to (overtly or covertly) exploit existing health inequities, by implying that scientific research and technological innovation have done little to resolve these and that solutions must lie elsewhere; or by implying that *not* using innovative therapies is preferable if accessibility to all cannot be guaranteed at the very outset. Whilst not all researchers are exclusively motivated by securing a better world for all – other motives exist - such conclusions often reflect a failure or an unwillingness to acknowledge the multiple sources of health inequities and the often slow, painstaking nature of progress in science, medicine and health policy. What is important now is that progress occurs on a trajectory towards greater health equity, with preclinical research doing its bit. Scientists should continue to defend their preclinical research efforts as one important element in the social enterprise of combatting the burden of RDs and other diseases. The suggestions made here might help them mount such a defence by fortifying the social licence to perform their research. We pre-clinical research scientists should discuss health equity and related social justice topics freely, but we also need institutional infrastructures, great and small, to promote, nurture and amplify the impact of such discussions.

## Data Availability

No datasets were generated or analysed during the current study.

## References

[CR1] Adachi T, El-Hattab AW, Jain R, Nogales Crespo KA, Lazo Q, Scarpa CI, Summar M, M., Wattanasirichaigoon D (2023) Enhancing Equitable Access to Rare Disease diagnosis and treatment around the World: a review of evidence, policies, and challenges. Int J Environ Res Public Health, 20 10.3390/ijerph20064732 PMC10049067 36981643

[CR2] Ambrosino E, Abou Tayoun AN, Abramowicz M, Zilfalil BA, Boughtwood T, Hamdi Y, Hubbard T, Kato K, Lopes-Cendes I, Majumder PP, Mascalzoni D, Ndiaye R, Ramsay M, Repetto GM, Shotelersuk V, Taylor S, Reeder JC, Ross AL (2024) The WHO genomics program of work for equitable implementation of human genomics for global health. Nat Med 30(10):2711-2713.10.1038/s41591-024-03225-x. 10.1038/s41591-024-03225-x 39227441

[CR3] Avery E, Clark J (2016) Sex-related reporting in randomised controlled trials in medical journals. Lancet 388:2839–2840 27979393 10.1016/S0140-6736(16)32393-5

[CR4] Baynam G, Molster C, Bauskis A, Kowal E, Savarirayan R, Kelaher M, Easteal S, Massey L, Garvey G, Goldblatt J, Pachter N, Weeramanthri TS, Dawkins HJS (2017) Indigenous Genetics and Rare diseases: Harmony, Diversity and Equity. Adv Exp Med Biol 1031:511–520 29214589 10.1007/978-3-319-67144-4_27

[CR5] Braveman P (2017) A New Definition Of Health Equity To Guide Future Efforts And Measure Progress. *Health Affairs 10.1377/forefront.20170622.060710* [Online]

[CR6] Carrot-Zhang J, Chambwe N, Damrauer JS, Knijnenburg TA, Robertson AG, Yau C, Zhou W, Berger AC, Huang KL, Newberg JY, Mashl RJ, Romanel A, Sayaman RW, Demichelis F, Felau I, Frampton GM, HAN S, Hoadley KA, Kemal A, Laird PW, Lazar AJ, Oak LEX, Shen N, Wong H, Zenklusen CK, Ziv JC, E., Beroukhim R (2020) Comprehensive Analysis of Genetic Ancestry and its molecular correlates in Cancer. Cancer Cell 37:639–654 e6 32396860 10.1016/j.ccell.2020.04.012 PMC7328015

[CR7] Gaviglio AM, Skinner MW, Finkel LOULJ, Augustine RS, E. F., Goldenberg AJ (2023) Gene-targeted therapies: towards equitable development, diagnosis, and access. Am J Med Genet C Semin Med Genet 193:56–63 36688577 10.1002/ajmg.c.32032

[CR8] Greenfield A (2017) Editing mammalian genomes: ethical considerations. Mamm Genome 28:388–393 28653171 10.1007/s00335-017-9702-y

[CR10] Halley MC, Smith HS, Ashley EA, Goldenberg AJ, Tabor HK (2022) A call for an integrated approach to improve efficiency, equity and sustainability in rare disease research in the United States. Nat Genet 54:219–222 35256804 10.1038/s41588-022-01027-w PMC9016354

[CR9] Halley MC, Halverson CME, Tabor HK, Goldenberg AJ (2023) Rare Disease, Advocacy and Justice: intersecting disparities in Research and Clinical Care. Am J Bioeth 23:17–26 37204146 10.1080/15265161.2023.2207500 PMC10321139

[CR11] Halmai NB, Carvajal-Carmona LG (2022) Diversifying preclinical research tools: expanding patient-derived models to address cancer health disparities. Trends Cancer 8:291–294 35125330 10.1016/j.trecan.2022.01.007 PMC11586084

[CR12] Harlow EM, Adair JE (2024) Make gene therapies more available by manufacturing them in lower-income nations. Nature 631:502–504 39020038 10.1038/d41586-024-02310-y

[CR13] Kleiderman E, Boardman F, Newson AJ, Laberge AM, Knoppers BM, RAVITSKY V (2024) Unpacking the notion of serious genetic conditions: towards implementation in reproductive decision-making? Eur J Hum Genet 10.1038/s41431-024-01681-0. 10.1038/s41431-024-01681-0 PMC11840117 39127803

[CR14] Kliegman M, Zaghlula M, Abrahamson S, Esensten JH, Wilson R, Urnov FD, Doudna JA (2024) A roadmap for affordable genetic medicines. Nature 10.1038/s41586-024-0788-7. 10.1038/s41586-024-07800-7 39019069

[CR15] Koblan LW, Erdos MR, Wilson C, Cabral WA, Levy JM, Xiong ZM, Tavarez UL, Davison LM, Gete YG, Mao X, Newby GA, Doherty SP, Narisu N, Sheng Q, Krilow C, Lin CY, Gordon LB, Cao K, Collins FS, Brown JD, Liu DR (2021) In vivo base editing rescues Hutchinson-Gilford progeria syndrome in mice. Nature 589:608–614 33408413 10.1038/s41586-020-03086-7 PMC7872200

[CR16] Lancet (2024) The fertility industry: profiting from vulnerability. Lancet 404:215 39032996 10.1016/S0140-6736(24)01484-3

[CR17] Liu H, Mei F, Ye R, Han X, Wang S, Ding Y, Zhi Y, Pang K, Guo W, Lu B (2024) APOE3ch alleviates Abeta and tau pathology and neurodegeneration in the human APP(NL-G-F) cerebral organoid model of Alzheimer’s disease. Cell Res 34:451–454 38609581 10.1038/s41422-024-00957-w PMC11143179

[CR18] Macias-Konstantopoulos WL, Collins KA, Diaz R, Duber HC, Edwards CD, Hsu AP, Ranney ML, Riviello RJ, Wettstein ZS, Sachs CJ (2023) Race, Healthcare, and Health disparities: a critical review and recommendations for advancing Health Equity. West J Emerg Med 24:906–918 37788031 10.5811/westjem.58408 PMC10527840

[CR19] Meng X, Yao D, Imaizumi K, Chen X, Kelley KW, Reis N, Thete MV, Mckinney A, Kulkarni A, Panagiotakos S, Bassik G, M. C., Pasca SP (2023) Assembloid CRISPR screens reveal impact of disease genes in human neurodevelopment. Nature 622:359–366 37758944 10.1038/s41586-023-06564-w PMC10567561

[CR20] Nair RR, Corrochano S, Gasco S, Tibbit C, Thompson D, Maduro C, Ali Z, Fratta P, Arozena AA, Cunningham TJ, Fisher EMC (2019) Uses for humanised mouse models in precision medicine for neurodegenerative disease. Mamm Genome 30:173–191 31203387 10.1007/s00335-019-09807-2 PMC6759662

[CR22] National Academy of Medicine, National Academy of Sciences and the Royal Society (2020) Heritable Human Genome Editing. Washington (DC). The National Academies Press. 10.17226/25665 32897669

[CR21] National Academies of Sciences, Engineering and Medicine (2017) Communities in Action: pathways to Health Equity. National Academies, Washington (DC). 10.17226/24624 28418632

[CR23] Nguengang Wakap S, Lambert DM, Olry A, Rodwell C, Gueydan C, Lanneau V, Murphy D, Le Cam Y, Rath A (2020) Estimating cumulative point prevalence of rare diseases: analysis of the Orphanet database. Eur J Hum Genet 28:165–173 31527858 10.1038/s41431-019-0508-0 PMC6974615

[CR24] Patwardhan V, Gil GF, Arrieta A, Cagney J, Degraw E, Herbert ME, Khalil M, Mullany EC, O’connell EM, Spencer CN, Stein C, Valikhanova A, Gakidou E, Flor LS (2024) Differences across the lifespan between females and males in the top 20 causes of disease burden globally: a systematic analysis of the global burden of Disease Study 2021. Lancet Public Health 9:e282–e294 38702093 10.1016/S2468-2667(24)00053-7 PMC11080072

[CR25] Reichart D, Newby GA, Wakimoto H, Lun M, Gorham JM, Curran JJ, Raguram A, Delaughter DM, Conner DA, Marsiglia JDC, Kohli S, Chmatal L, Page DC, Zabaleta N, Vandenberghe L, LIU, D. R., SEIDMAN, J. G., SEIDMAN C (2023) Efficient in vivo genome editing prevents hypertrophic cardiomyopathy in mice. Nat Med 29:412–421 36797483 10.1038/s41591-022-02190-7 PMC9941048

[CR26] Rothstein MA, Zimmerer KC, Andanda P, Arawi T, Arzuaga F, Chen H, de Vries M, Dove ES, Ghaly M, Hatanaka R, Hendriks AC, Hernandez MC, Ho CWL, Joly Y, Krekora-Zajac D, Lee WB, Mattsson T, Molnar-Gabor F, Namalwa K, Nicolas P, Nielsen J, Nnamuchi O, Otlowski M, Palmour N, Rial-Sebbag E, Siegal G, Wathuta JM, Zawati MH, Knoppers BM (2024) International scope of biomedical research ethics review. Science 385:145–147 38991077 10.1126/science.adp6277

[CR27] Sergouniotis PI, Fitzgerald T, Birney E (2023) From genetic variation to precision medicine. Camb Prism Precis Med 1:e7 38550939 10.1017/pcm.2022.11 PMC10953743

[CR28] Serrano JG, O’Leary M, Vannoy GE, Mangilog BE, Holm IA, Fraiman YS, Rehm HL, O’donnell-Luria A, Wojcik MH (2023) Advancing understanding of inequities in Rare Disease Genomics. Clin Ther 45:745–753 37517917 10.1016/j.clinthera.2023.06.010 PMC10527807

[CR29] Shaaban M, Duut A, Mensah N (2024) We are junior scientists from emerging economies - the world needs more researchers like us solving global problems. Nature 10.1038/d41586-024-24854 10.1038/d41586-024-02485-4 39060530

[CR30] Singh A, Irfan H, Fatima E, Nazir Z, Verma A, Akilimali A (2024) Revolutionary breakthrough: FDA approves CASGEVY, the first CRISPR/Cas9 gene therapy for sickle cell disease. Ann Med Surg (Lond) 86:4555–4559 39118728 10.1097/MS9.0000000000002146 PMC11305803

[CR31] Tkemaladze T (2024) Mining the diagnosis of rare disease with limited resources. Nat Genet 56:1323 38965416 10.1038/s41588-024-01818-3

[CR32] WHO (2023) Infertility prevalence estimates, 1990–2021. Geneva

[CR33] Zernicka-Goetz M (2023) The evolution of embryo models. Nat Methods 20:1844–1848 38057512 10.1038/s41592-023-02077-6

